# The prevalence of hypertension and its distribution by sociodemographic factors in Central Mozambique: a cross sectional study

**DOI:** 10.1186/s12889-020-09947-0

**Published:** 2020-12-01

**Authors:** Matsuzaki Mika, Sherr Kenneth, Augusto Orvalho, Kawakatsu Yoshito, Ásbjörnsdóttir Kristjana, Chale Falume, Covele Alfredo, Manaca Nelia, Muanido Alberto, Bradley H. Wagenaar, Ana O. Mocumbi, Gimbel Sarah, the InCoMaS Study Team, Joao Luis Manuel, Leecreesha Hicks, Arlete Mahumane, James Pfeiffer, Stephen Gloyd, Fatima Cuembelo, Miguel Nhumba, Joaquim Lequechane, Manuel Napua, Lucia Vieira, Alberto Muanido, Cathy Michel

**Affiliations:** 1 Department of International Health, Johns Hopkins Bloomberg School of Public Health, Baltimore, MD USA; 2 Department of Global Health, University of Washington, Seattle, WA USA; 3 Health Alliance International, Seattle, WA USA; 4 Department of Community Medicine, Eduardo Mondlane University, Maputo, Mozambique; 5 Department of Epidemiology, University of Washington, Seattle, WA USA; 6 CIOB, University of Washington, Seattle, WA USA; 7 INS, Maputo, Mozambique; 8 Department of Family and Child Nursing, University of Washington, Seattle, WA USA

**Keywords:** Hypertension, Low and middle income countries, Mozambique, Nutrition transition

## Abstract

**Background:**

Hypertension (HTN) is a major risk factor for cardiovascular diseases, and its prevalence has been rising in low- and middle-income countries. The current study describes HTN prevalence in central Mozambique, association between wealth and blood pressure (BP), and HTN monitoring and diagnosis practice among individuals with elevated BP.

**Methods:**

The study used data from a cross-sectional, representative household survey conducted in Manica and Sofala provinces, Mozambique. There were 4101 respondents, aged ≥20 years. We measured average systolic and diastolic BP (SBP and DBP) from three measurements taken in the household setting. Elevated BP was defined as having either SBP ≥140 or DBP ≥90 mmHg.

**Results:**

The mean age of the participants was 36.7 years old, 59.9% were women, and 72.5% were from rural areas. Adjusting for complex survey weights, 15.7% (95%CI: 14.0 to 17.4) of women and 16.1% (13.9 to 18.5) of men had elevated BP, and 7.5% (95% CI: 6.4 to 8.7) of the overall population had both SBP ≥140 and DBP ≥90 mmHg. Among participants with elevated BP, proportions of participants who had previous BP measurement and HTN diagnosis were both low (34.9% (95% CI: 30.0 to 40.1) and 12.2% (9.9 to 15.0) respectively). Prior BP measurement and HTN diagnosis were more commonly reported among hypertensive participants with secondary or higher education, from urban areas, and with highest relative wealth. In adjusted models, wealth was positively associated with higher SBP and DBP.

**Conclusions:**

The current study found evidence of positive association between wealth and BP. The prevalence of elevated BP was lower in Manica and Sofala provinces than the previously estimated national prevalence. Previous BP screening and HTN diagnosis were uncommon in our study population, especially among rural residents, individuals with lower education levels, and those with relatively less wealth. As the epidemiological transition advances in Mozambique, there is a need to develop and implement strategies to increase BP screening and deliver appropriate clinical services, as well as to encourage lifestyle changes among people at risk of developing hypertension in near future.

**Supplementary Information:**

**Supplementary information** accompanies this paper at 10.1186/s12889-020-09947-0.

## Background

Cardiovascular diseases (CVD) contribute to the highest number of premature deaths globally [1], and hypertension (HTN) is one of its leading causes [2]. As low- and middle-income countries (LMIC) develop economically, nutrition and lifestyle patterns are changing, and many countries are following the footsteps of high income countries during the epidemiological transition. In lower income countries, non-communicable disease (NCD) epidemics are generally seen first among wealthier individuals, though economic development and globalization eventually bring obesogenic diets and other NCD risk factors to more disadvantaged populations [3–5].

The World Health Organization estimated that the prevalence of HTN may be highest in the Africa region compared to other regions in the world, where 46% of adults aged 25 and above are estimated to have hypertension [6]. In sub-Saharan Africa (SSA), a systematic review showed the pooled prevalence estimate of HTN among ≥15 year olds to be around 30% (95% confidence interval (CI): 27 to 34) [7]. Among older adults (> 50 years old) in SSA, a recent systematic review reported a disconcertingly high estimate for the overall pooled prevalence at 57.0% (95% CI 52–61%) [8]. In SSA, it is estimated that HTN accounts for 52.5% of all stroke [9]. The Pan-African Society of Cardiology (PASCAR) identified addressing HTN as the highest priority in reducing CVD in Africa [10]. PASCAR recognizes that – despite the rising prevalence of HTN – awareness of hypertensive status is low in this region. A recent national study showed that the prevalence of hypertension increased from 33.1 to 38.9% between 2005 and 2015 in Mozambique, but awareness of hypertensive status (13.8% in 2005 and 13.5% in 2015) as well as treatment among those aware (51.9% in 2005 and 50.1% in 2015) remained unchanged [11, 12]. There is a need to address both the increase in incidence of HTN as well as clinical management of existing HTN cases.

As economic development is occurring unevenly in Mozambique, it is important to assess current status of nutrition and epidemiological transition by region. In fact, the aforementioned national study in Mozambique observed that hypertension was more common among certain sub-populations, such as women living in urban areas. Analyses disaggregated by sociodemographic and economic subgroups are useful as different subpopulations are experiencing various stages of nutritional and epidemiological transitions. Identification of the current status of these transitions can guide development of strategies tailored towards the needs of each subpopulation.

The objective of this paper is to describe prevalence of elevated BP as well as previous BP screening and hypertension diagnosis - with consideration to relative wealth status - among a representative sample of households in central Mozambique. We aimed to identify specific individual- and community-level factors associated with hypertension, BP screening, and hypertension disagnosis that can motivate targeted interventions.

## Methods

### Study design

The Inquérito Comunitário de Manica e Sofala (InCoMas) was a cross-sectional community survey conducted to provide endline data for an impact evaluation of a seven-year health system strengthening intervention in Sofala and Manica provinces [13]. A detailed description of the sampling procedures has been previously published [14]. Briefly, the study collected data from households in Manica and Sofala provinces from September 2016 to February 2017. As recent census data were not available in Mozambique, we created a sampling frame of buildings from available satellite imagery to generate a primary sampling unit of a square on a grid of 2.106 km (0.02 decimal degrees). From this frame, a random sample with probabilities proportional to size was drawn. A total of 3087 households were visited (1549 in Sofala and 1538 in Manica), a standardized set of questionnaires applied, and BP measurements collected for eligible members in each household. There was only one instance of survey refusal [14]. The current analyses include adults aged 20 years and older. This study was approved by the Institutional Review Board of the National Institute of Health in Mozambique (IRB00002657). The ethical approvals were in consideration of Helsinki, as noted in the IRB approval letter. Written informed consent was obtained from all participants.

### Measurements

#### Questionnaire data

A structured questionnaire was administered to all participants by trained interviewers. The survey questions related to the variables in this analysis have been translated into English and included in the Supplemental Material S3. We collected information on self-reported demographic characteristics (age, gender, education) as well as current lifestyles (smoking and alcohol use). Education levels were categorized into 3 levels: primary school or less, secondary school, and above secondary school. Alcohol use was categorized into 3 levels: never, infrequent (twice a month or less), frequent (more than twice a week). Participants were also asked whether they have ever had their BP measured, whether they have been diagnosed with hypertension, and whether they have ever had a heart attack, chest pain due to heart diseases, or a stroke. Urban or rural residence was assigned using the data from the Mozambique National Institute of Statistics.

Wealth index was calculated using the method described in the Demographic and Health Survey Wealth Index, with a modification to the individual items included in the Principle Component Analysis [15]. We used the following items from the household survey: household characteristics (type of floor, roof, latrine, source of drinking water and main cooking energy) and ownership of household items (radio, TV, bed, fridge, bicycle, motorcycle, car, boat, mobile phone and landline phone). Wealth quintiles were classified into 3 groups to indicate relative wealth: below average (1st and 2nd quintiles), average (3rd quintile), and above average (4th and 5th quintiles).

#### Anthropometric and clinical data

Weight was measured on NOVA Electronic Personal Glass Scale BGS-1240 to the nearest 100 g and height was measured to the nearest 10 mm. Measurements were taken once. Body mass index (BMI) was calculated as weight (kg)/height(m^2^). Systolic and diastolic BP (SBP and DBP) were measured on Orbis Pharma BP-1305 three times: first following 5 min of rest at the beginning of the survey, with second and third measurements following 10–15 min intervals between measurements. The average of the first two measurement values was used as the final BP estimate for each individual unless they differed by >10mmg, in which case we used the second and third measurement values [16]. Participants with only one valid measurement was removed from the analyses. Though there has been a recent change in the definition of hypertension by American College of Cardiology and American Hearts Association (ACC/AHA; SBP ≥ 130 mmHg or DBP ≥ 80), [17] the current study used SBP ≥ 140 or DBP ≥ 90 as the cutoff values for *elevated BP* to improve comparability to previous studies in Mozambique and other LMIC.

### Statistical analysis

Descriptive statistics were calculated for all adult participants by gender and with and without adjustment with complex sampling weights. The proportion of participants with elevated BP who 1) had ever had their BP measured and 2) had ever been diagnosed with hypertension were also calculated by age groups, gender, and education levels with and without adjustment with complex sampling weights. Both means (for body mass index (BMI) and systolic and diastolic BP) and proportions of hypertension were estimated accounting for the complex sampling design. Variance for proportions was estimated with *svyciprop* in R. We used weights as the inverse probability of selection into the sample. Listwise deletion was performed for missing data.

Associations between systolic and diastolic BPs and relative wealth were assessed using random-intercept multilevel models accounting for household level differences. Model 1 and 3 were adjusted for age, gender, BMI, urbanicity, education level, and province. Model 2 and 4 were further adjusted for relative wealth. All analyses were done in R (version 3.4.4). We included the results from logistic regression models with an outcome of having elevated BP (SBP ≥ 140 mmHg or DBP ≥ 90) in Supplemental Table S1 and with an outcome using ACC/AHA’s definition of hypertension (SBP ≥ 130 mmHg or DBP ≥ 80) in Supplemental Table S2.

## Results

Among 4101 participants aged 20 or above, the mean age was 36.7 years old, 59.9% were women, and 72.5% were from rural areas (Table 1).

**Table 1:** Sociodemographic and behavioral characteristics of the adult participants (≥20 years old) in the InCoMas

	All			Men			Women		
	*n*	Unadjusted %	Adjusted % (95% CI)	*n*	Unadjusted %	Adjusted % (95% CI)	*n*	Unadjusted %	Adjusted % (95% CI)
	4101			1643			2458		
Age groups									
20 to < 30	1516	37	36.9 (34.5 to 39.4)	453	27.6	27.2 (24.9 to 29.6)	1063	43.2	43.3 (40.2 to 46.6)
30 to < 40	1167	28.5	28.8 (27.1 to 30.5)	483	29.4	29.8 (27.3 to 32.5)	684	27.8	28 (26.0 to 30.1)
40 to < 50	688	16.8	16.7 (15.3 to 18.1)	337	20.5	20.7 (18.8 to 22.9)	351	14.3	13.9 (12.4 to 15.7)
50 to < 60	406	9.9	9.6 (8.6 to 10.7)	196	11.9	11.5 (9.9 to 13.2)	210	8.5	8.3 (7.2 to 9.7)
60 and above	324	7.9	8.1 (7.0 to 9.3)	174	10.6	10.7 (9.1 to 12.6)	150	6.1	6.3 (5.2 to 7.6)
Residence									
urban	1125	27.5	21.3 (15.9 to 28)	469	28.7	21.4 (15.7 to 28.3)	656	26.8	21.3 (15.8 to 28.1)
rural	2961	72.5	78.7 (72.0 to 84.1)	1167	71.3	78.6 (71.7 to 84.3)	1794	73.2	78.7 (71.9 to 84.2)
Education									
primary or less	2157	68.3	70.4 (66.3 to 74.1)	906	61.9	64.3 (59.9 to 68.5)	1251	73.8	75.5 (70.9 to 79.7)
secondary	878	27.8	26.3 (23.3 to 29.6)	473	32.3	30.7 (27.4 to 34.3)	405	23.9	22.6 (18.9 to 26.7)
above secondary	125	4	3.3 (2.3 to 4.6)	85	5.8	4.9 (3.5 to 7.0)	40	2.4	1.9 (1.2 to 2.8)
Relative wealth									
below average	1716	42	45.7 (39.7 to 51.7)	687	41.9	46.3 (39.9 to 52.9)	1029	42	45.2 (39.3 to 51.3)
average	832	20.3	22.0 (19.3 to 24.9)	326	19.9	21.9 (18.9 to 25.2)	506	20.7	22.0 (19.2 to 25.1)
above average	1542	37.7	32.4 (26.2 to 39.2)	627	38.2	31.8 (25.4 to 38.9)	915	37.3	32.8 (26.5 to 39.7)
Alcohol use									
never	2052	75.5	75.8 (73.2 to 78.3)	785	66.8	66.0 (62.4 to 69.5)	1267	82.2	83.1 (80.3 to 85.7)
infrequent	526	19.4	19.1 (17.0 to 21.3)	299	25.4	25.8 (22.9 to 29)	227	14.7	14.0 (11.8 to 16.6)
frequent	140	5.2	5.1 (4.2 to 6.2)	92	7.8	8.1 (6.4 to 10.2)	48	3.1	2.8 (2.0 to 4.0)
Smoking									
yes	82	3	3.0 (2.3 to 3.8)	54	4.1	4.0 (2.9 to 5.5)	28	1.9	2.0 (1.3 to 3.0)
no	2683	97	97.0 (96.2 to 97.7)	1255	95.9	96.0 (94.5 to 97.1)	1428	98.1	98.0 (97.0 to 98.7)
CVD ever									
yes	194	7	6.8 (5.6 to 8.2)	84	6.4	6.1 (4.7 to 7.8)	110	7.6	7.4 (5.9 to 9.3)
no	2556	92.4	92.5 (91.0 to 93.8)	1218	93	93.1 (91.4 to 94.5)	1338	91.9	92.0 (90.0 to 93.6)
don’t know	16	0.6	0.7 (0.4 to 1.1)	8	0.6	0.8 (0.4 to 1.6)	8	0.5	0.6 (0.3 to 1.1)

All participants included in this table had age, sex, systolic blood pressure, and diastolic blood pressure by province. Adjusted % (95% confidence interval) accounts for sampling weights

*CI* confidence interval, *CVD* cardiovascular diseases

Most participants had attained primary school education or less (68.3%). A higher proportion of men than women had attained higher than primary school education. There were very few people who continued onto education above secondary school for either gender. Neither frequent alcohol use nor smoking was commonly reported in this population. Only 7% of the participants reported having had cardiovascular diseases in the past.

Adjusting for complex survey weights, 15.7% (95%CI: 14.0 to 17.4) of women and 16.1% (13.9 to 18.5) of men had elevated BP, and 7.5% (95% CI: 6.4 to 8.7) of the overall population had both SBP ≥140 and DBP ≥90. Table 2 shows that the weighted averages were 121.3 mmHg (95% CI: 120.2 to 122.4) for SBP and 76.0 mmHg (75.2 to 76.8) for DBP. Across all three measures, we saw higher values in more wealthy groups.

**Table 2:** Weighted means for body mass index and systolic and diastolic blood pressure by wealth index

Wealth index group	All		Below average		Average		Above average	
	Mean	95% CI	Mean	95% CI	Mean	95% CI	Mean	95% CI
**BMI (m**^**2**^**/kg)**	21.8	21.6 to 22.0	20.9	20.7 to 21.1	21.7	21.4 to 22.0	23.0	22.7 to 23.3
**SBP (mmHg)**	121.3	120.2 to 122.4	119.7	118.2 to 121.2	121.5	119.3 to 123.8	123.3	121.6 to 124.9
**DBP (mmHg)**	76.0	75.2 to 76.8	74.0	73.0 to 75.0	76.2	74.8 to 77.5	78.6	77.5 to 79.6

*CI* confidence interval, *BMI* body mass index, *SBP* systolic blood pressure, *DBP* diastolic blood pressure

Wealth index groups were defined as: Below average = bottom two quintiles; Average = middle quintiles; Above average = top two quintiles

Overall, previous BP measurement and diagnosis were low among participants with elevated BP (SBP ≥ 140 or DBP ≥ 90): less than 40% of the participants with elevated BP reported having ever had their BP measured and the proportion having received HTN diagnosis in the past was even less at 14.2% (Table 3).

**Table 3:** Prior experience with blood pressure measurements and hypertension diagnosis among participants with elevated blood pressure

	Total*	BP previously measured			HTN previously diagnosed		
		*n*	Unadjusted % within each subgroup	Adjusted % (95% CI)	*n*	Unadjusted % within each subgroup	Adjusted % (95% CI)
**All**	513	204			73		
**Age groups**							
**20 to < 30**	121	49	40.5	36.2 (27.8 to 45.5)	18	14.9	13.6 (8.3 to 21.5)
**30 to < 40**	134	69	51.5	45.9 (35.9 to 56.2)	24	18	15.5 (10.2 to 22.8)
**40 to < 50**	88	36	40.9	34.1 (23.4 to 46.7)	13	14.8	12.7 (7.2 to 21.4)
**50 to < 60**	83	26	31.3	28.1 (18.6 to 40)	11	13.3	11.4 (5.8 to 21.2)
**60 and above**	88	24	27.3	23.3 (14.9 to 34.6)	7	8	5.8 (2.8 to 11.9)
**Gender**							
**Men**	246	94	38.2	33 (26.5 to 40.3)	35	14.3	12.7 (9 to 17.6)
**Women**	268	110	41	36.5 (30.3 to 43.2)	38	14.2	11.8 (8.7 to 15.8)
**Residence**							
**Urban**	197	112	56.9	52.2 (42.9 to 61.3)	45	22.8	21.3 (16.9 to 26.5)
**Rural**	313	90	28.8	27.5 (22.8 to 32.8)	28	8.9	8.6 (6.3 to 11.5)
**Education level**							
**Primary or less**	245	80	32.7	28.4 (22.5 to 35)	29	11.8	9.6 (6.4 to 14.1)
**Secondary**	111	71	64	58.3 (48 to 68)	28	25.2	24.6 (17.3 to 33.8)
Above secondary**	24	14	58.3	54.4 (28.7 to 77.9)	7	29.2	26.4 (10.5 to 52.4)
**Relative wealth**							
**below average**	163	40	24.5	23.9 (18.1 to 30.9)	12	7.4	7.6 (4.6 to 12.4)
**average**	91	25	27.5	28.2 (20.5 to 37.4)	5	5.5	5.5 (2.3 to 12.5)
**above average**	259	139	53.7	47.9 (40.9 to 55.1)	56	21.7	19.6 (15.8 to 24.1)

Elevated blood pressure was defined as systolic blood pressure > =140 or diastolic blood pressure > =90. Adjusted % (95%CI) accounts for sampling weights

*BP* blood pressure, *HTN* hypertension, *CI* confidence interval

* Total includes those who answered “don’t know” to prior blood pressure measurements and hypertension diagnosis, in addition to those who answers “Yes” or “No”

* * “Above secondary” includes technical schools

Generally, greater percentages of younger participants with elevated BP reported having received previously BP measurements or the diagnosis previously although there were proportionally fewer people from the youngest group (20–29 years old) reporting previous BP measurements or previous diagnosis than the 30–39 year olds. Slightly greater percentages of women had previous BP measurement, possibly due to routine measurements conducted during prenatal care. Prior diagnosis was more common among participants with secondary or higher education as well as among urban residents than participants with less education and rural residents respectively. Awareness and diagnosis were higher in the wealthiest group than the average or low relatively wealth groups.

In the multilevel models, relatively higher wealth (above average) was associated with higher SBP and DBP (Table 4).

**Table 4:** Multi-level models examining association of relative wealth and 1) systolic blood pressure and 2) diastolic blood pressure

	Systolic blood pressure				Diastolic blood pressure			
	Model 1	*p*	Model 2	*p*	Model 3	*p*	Model 4	*p*
	*n* = 2190		*n* = 2185		*n* = 2190		*n* = 2185	
	β (95% CI)		β (95% CI)		β (95% CI)		β (95% CI)	
**Rural residence**	−3.97 (−5.62 to −2.32)	< 0.001	−1.48 (− 3.46 to 0.5)	0.144	−4.08 (−5.25 to − 2.9)	< 0.001	−1.93 (− 3.33 to −0.53)	0.007
**Age**								
**30–39**	1.36 (− 0.47 to 3.2)	0.146	1.16 (− 0.67 to 2.99)	0.214	2.87 (1.57 to 4.17)	< 0.001	2.69 (1.4 to 3.99)	< 0.001
**40–49**	4.49 (2.17 to 6.8)	< 0.001	4.2 (1.89 to 6.51)	< 0.001	4.76 (3.12 to 6.41)	< 0.001	4.61 (2.98 to 6.25)	< 0.001
**50–59**	10.09 (7.18 to 13)	< 0.001	9.88 (6.98 to 12.78)	< 0.001	6.08 (4.01 to 8.15)	< 0.001	5.87 (3.82 to 7.93)	< 0.001
**60 and above**	17.64 (14.23 to 21.05)	< 0.001	17.4 (14 to 20.79)	< 0.001	8.82 (6.4 to 11.24)	< 0.001	8.62 (6.22 to 11.02)	< 0.001
**Gender (women)**	−1.61 (− 3.21 to − 0.01)	0.049	− 2.22 (− 3.83 to − 0.61)	0.007	0.15 (− 0.99 to 1.28)	0.801	−0.41 (− 1.55 to 0.73)	0.482
**BMI (kg/m**^**2**^**)**	0.69 (0.49 to 0.89)	< 0.001	0.61 (0.41 to 0.82)	< 0.001	0.49 (0.35 to 0.63)	< 0.001	0.42 (0.28 to 0.56)	< 0.001
**Education**								
**secondary**	− 0.50 (− 2.27 to 1.28)	0.583	−1.8 (− 3.65 to 0.06)	0.058	1.37 (0.11 to 2.63)	0.034	0.18 (− 1.14 to 1.49)	0.791
**above secondary**	0.96 (−2.89 to 4.81)	0.626	−0.83 (− 4.75 to 3.1)	0.68	2.96 (0.22 to 5.7)	0.034	1.35 (− 1.42 to 4.13)	0.341
**Relative wealth**								
**below average**			−1.68 (−3.78 to 0.42)	0.117			−2.43 (−3.92 to − 0.95)	0.001
**above average**			3.41 (1.09 to 5.72)	0.004			2.33 (0.69 to 3.97)	0.005

All models were adjusted for province as a covariate and accounted for household-level differences in multilevel models

The reference group was: 20–29 year olds for age groups; urban areas for residence; men for gender; the middle quintile (average) for wealth; below secondary for education

Lower wealth (below average) was negatively associated with BP although the evidence for this pattern was weaker for SBP (β: -1.68; 95% CI: − 3.78 to 0.42) than DBP (− 2.43; − 3.92 to − 0.95). Both SBP and DBP were positively associated with urban residence; however, this association was attenuated when relative wealth group was added to the models. There was a small positive association between BMI and blood pressure. Alcohol use and smoking status were also examined but there was no clear evidence of association in this study population (data not shown). The logistic regression models with an outcome of elevated BP (SBP ≥ 140 mmHg or DBP ≥ 90 mmHg) showed similar patterns of associations between the outcome and relative wealth group although the strengths of evidence were weaker (Odds Ratio: 1.47 (95% CI: 1.00 to 2.15) for above average; 0.81 (0.55 to 1.17) for below average, relative to the average wealth group) (Supplemental Table S1). When the more stringent ACC/AHA guideline for elevated BP (SBP ≥ 130 mmHg or DBP ≥ 80 mmHg) was used in the same logistic models, there was stronger evidence for the positive association between wealth and elevated BP (Supplemental Table S2).

## Discussion

The current study found that about 16% of the population in Manica and Sofala provinces had elevated BP, which was lower than the national estimate by a previous study [12]. There was clear evidence of a positive association between relative wealth and BP in this population in central Mozambique. Overall, less than half of the individuals with elevated BP had prior exposure to BP measurements or HTN diagnoses. Both prior BP measurement and HTN diagnosis were more common in younger participants, among urban residents, and in wealthier groups. As Mozambique continues its economic development, it is important to start implementing proper clinical management and awareness education to change the trajectory towards the epidemic of hypertension.

Nutritional and epidemiological transitions – from undernutrition and infectious diseases to obesity and NCD epidemics – typically occur first amongst wealthier populations in settings undergoing economic development [18–20]. Wealthier populations have also been the first to reach a point of behavioral changes to move towards healthier lifestyles in higher income countries, resulting in an epidemiological transition where the burden of NCD lies more heavily on disadvantaged populations. In the United States, CVD risk factors like obesity and diabetes mellitus II are now more common in populations with lower education and income levels [21, 22].

Hypertension is of particular importance to public health officials around the world, due to its association with a number of diseases contributing to premature deaths. Along with rapid economic development, hypertension prevalence have increased dramatically in many LMIC, including Mexico, South Africa, and China [23]. Studies have found that both country-level economic development and individual socioeconomic status play important roles in the epidemiological transition [24]. Association of individual socioeconomic status and hypertension vary by the levels of national economic development and inequity as well as by urbanicity within countries [25, 26]. Countries in sub-Saharan Africa are at various stages of economic development and as a result, associations between wealth and hypertension in sub-Saharan Africa have shown conflicting results. For example, in studies from urban Ghana, South Africa, and urban Tanzania, hypertension was high even among the lowest quintile group, [27, 28] while studies from Nigeria and Kenya found patterns of greater prevalence of hypertension among wealthier groups, similar to our findings [29–32].

Given our study results on the current status of association between wealth and hypertension in Mozambique, there is a need to both prevent future epidemics in a population who have not developed hypertension and also to help maintain lower BP in hypertensive individuals. Even though there are some overlapping strategies for both populations, in countries where dual burden of malnutrition exist, additional consideration is needed for those who suffer from undernutrition. Public health officials may want to explore distinct strategies for those individuals to achieve a healthier nutrition transition as they recover from undernutrition, in addition to implementing strategies to increase awareness of hypertension and promote healthier lifestyles in the overall population.

In both prevention and treatment of hypertension, strengthening of health care systems in Mozambique is crucial as they affect both BP monitoring and diagnose/treatment of hypertension. In our prior assessment of health system determinants of BP screening for patients accessing health care, we found that inconsistent BP measurements were associated with chronic stock outs of materials, equipment and medicines. In a separate study we are conducting, our preliminary data showed that – while most health facilities have at least one basic equipment to assess BP – often the facilities have only one piece of equipment that is shared among all clinic services including antenatal care, maternities, outpatient care, and emergency departments. This type of limitation of basic resources ought to be addressed.

Finally, a life-course approach to hypertension prevention may benefit settings like Mozambique, where large segments of the population experienced undernutrition in early life. In 2008/2009, the prevalence of underweight, stunting, and wasting were 19, 48, and 7% respectively in Mozambique [33]. Previous research suggests that individuals who experienced nutritional problems in early life may be more susceptible to experiencing NCD in later life [34]. Future research is needed to develop optimal strategies to address additional impact of adverse conditions in early life on adult-onset diseases like hypertension in low-income countries experiencing the epidemiological transitions.

### Strength and limitations

This study was able to estimate a region-specific prevalence of hypertension, using a unique sampling method in areas where updated census data are not available. Given a small number of studies examining the association between hypertension and socioeconomic status in sub-Saharan Africa, the current study contributes insight on the epidemiological transition in sub-Saharan Africa. The survey included both urban and rural areas, allowing us to show differences in prevalence of hypertension within the country. The availability of wealth index strengthened the analysis as it highlighted its potential role in the association between urbanicity and BP. Our survey also probed BP measurement and hypertension diagnosis practices, adding further evidence on problems associated with health care delivery. Despite these strengths, the cross-sectional nature of the study limits causal inference on wealth and elevated BP. We determined the hypertension status based on one clinical visit due to the study limitation but ideally, at least two separate visits should be used to clinically diagnose hypertension. We were also unable to cover all areas of these two provinces due to issues with armed conflict and therefore the results are not fully representative at the provincial level. The proportion of the women who participated in this study were slightly higher (59.9%) than – though comparable to – the latest census from 2017 (53.7% in Manica and 52.7% in Sofala). In Mozambique, many male heads-of -household work outside of their community of origin, often being away from their home for long periods of time. In addition, a household had children and mothers present, we went through modules on children and maternal health first and, by the end of the interview, men may have drifted away, which may have led to a slightly higher representation of women in this study. Furthermore, InCoMas did not collect data on HIV status due to potential loss of trust by the respondents, which may have introduced bias on the association between urbanicity and elevated BP.

## Conclusion

We saw evidence of a positive association between wealth and BP in the Manica and Sofala provinces. BP monitoring and diagnosis of hypertension were not common among participants with elevated BP, especially among rural residents, individuals with lower education levels, older age groups, and less wealthy groups. As the epidemiological transition progresses, there is a need to implement strategies to increase awareness of the importance of hypertension prevention and care, and also to improve BP monitoring across all socioeconomic strata in central Mozambique.

## Supplementary Information

**Additional file 1:** **Supplemental Material S3.** Relevant sections from the InCoMaS 2016 Community Survey Manica and Sofala: (English version).

**Additional file 2:** **Table S1.** Cross-sectional associations of elevated blood pressure (systolic blood pressure ≥ 140 mmHg or diastolic blood pressure ≥ 90 mmHg) with risk factors. **Table S2.** Cross-sectional association of hypertension with risk factors using American Heart Association’s definition (SBP ≥ 130 mmHg or DBP ≥ 80 mmHg).

## Data Availability

Access to data may be requested from the InCoMaS team at hai@uw.edu.

## Abbreviations

HTN: Hypertension
BP: Blood Pressure
SBP: Systolic blood pressure
DBP: Diastolic blood pressure
CI: Confidence interval
CVD: Cardiovascular disease
LMIC: Low and middle income countries
NCD: Non-communicable disease
SSA: Sub-Saharan Africa
PASCAR: Pan-African Society of Cardiology
InCoMas: The Inquérito Comunitário de Manica e Sofala
BMI: Body mass index

## References

[1] Roth GA, Forouzanfar MH, Moran AE, Barber R, Nguyen G, Feigin VL (2015). Demographic and epidemiologic drivers of global cardiovascular mortality. N Engl J Med.

[2] Kannel WB (1996). Blood pressure as a cardiovascular risk factor: prevention and treatment. JAMA..

[3] Prentice AM (2006). The emerging epidemic of obesity in developing countries. Int J Epidemiol.

[4] Prentice A, Webb F (2006). Obesity amidst poverty. Int J Epidemiol.

[5] Leng B, Jin Y, Li G, Chen L, Jin N (2015). Socioeconomic status and hypertension: a meta-analysis. J Hypertens.

[6] World Health Organization. A global brief on hypertension. 2013. http://www.who.int/cardiovascular_diseases/publications/global_brief_hypertension/en/. Accessed 31 Jul 2018.

[7] Feven A, Sebhat E, Stephen K, Betiglu T, Echouffo-Tcheugui Justin B, Kengne Andre P (2015). Burden of Undiagnosed Hypertension in Sub-Saharan Africa. Hypertension.

[8] Bosu WK, Reilly ST, Aheto JMK, Zucchelli E (2019). Hypertension in older adults in Africa: a systematic review and meta-analysis. PLoS One.

[9] O’Donnell MJ, Chin SL, Rangarajan S, Xavier D, Liu L, Zhang H (2016). Global and regional effects of potentially modifiable risk factors associated with acute stroke in 32 countries (INTERSTROKE): a case-control study. Lancet.

[10] Dzudie A, Rayner B, Ojji D, Schutte AE, Twagirumukiza M, Damasceno A (2018). Roadmap to achieve 25% hypertension control in Africa by 2025. Glob Heart.

[11] Damasceno A, Azevedo A, Silva-Matos C, Prista A, Diogo D, Lunet N (2009). Hypertension prevalence, awareness, treatment, and control in mozambique: urban/rural gap during epidemiological transition. Hypertens Dallas Tex 1979.

[12] Jessen N, Damasceno A, Silva-Matos C, Tuzine E, Madede T, Mahoque R (2018). Hypertension in Mozambique: trends between 2005 and 2015. J Hypertens.

[13] Sherr K, Cuembelo F, Michel C, Gimbel S, Micek M, Kariaganis M (2013). Strengthening integrated primary health care in Sofala, Mozambique. BMC Health Serv Res.

[14] Wagenaar BH, Augusto O, Ásbjörnsdóttir K, Akullian A, Manaca N, Chale F (2018). Developing a representative community health survey sampling frame using open-source remote satellite imagery in Mozambique. Int J Health Geogr.

[15] Rustein SO. Steps to constructing the new DHS Wealth Index. https://www.dhsprogram.com/programming/wealth%20index/Steps_to_constructing_the_new_DHS_Wealth_Index.pdf. Accessed 31 Jul 2018.

[16] Williams B, Mancia G, Spiering W, Agabiti Rosei E, Azizi M, Burnier M (2018). 2018 ESC/ESH guidelines for the management of arterial hypertensionThe task force for the management of arterial hypertension of the European Society of Cardiology (ESC) and the European Society of Hypertension (ESH). Eur Heart J.

[17] Whelton PK, Carey RM, Aronow WS, Casey DE, Collins KJ, Cheryl DH (2018). 2017 ACC/AHA/AAPA/ABC/ACPM/AGS/APhA/ASH/ASPC/NMA/PCNA guideline for the prevention, detection, evaluation, and Management of High Blood Pressure in adults: a report of the American College of Cardiology/American Heart Association task force on clinical practice guidelines. Hypertension..

[18] Omran AR (1971). The epidemiologic transition. A theory of the epidemiology of population change. Milbank Mem Fund Q.

[19] Popkin BM (1993). Nutritional patterns and transitions. Popul Dev Rev.

[20] Popkin BM (2003). The nutrition transition in the developing world. Dev Policy Rev.

[21] Drewnowski A (2009). Obesity, diets, and social inequalities. Nutr Rev.

[22] Min YI, Anugu P, Butler KR, Hartley TA, Mwasongwe S, Norwood AF, Sims M, Wang W, Winters KP, Correa A (2017). Cardiovascular disease burden and socioeconomic correlates: findings from the Jackson Heart Study. J Am Heart Assoc..

[23] Sarki AM, Nduka CU, Stranges S, Kandala NB, Uthman OA. Prevalence of hypertension in low- and middle-income countries: a systematic review and meta-analysis. Medicine (Baltimore). 2015;94(50):e1959. 10.1097/MD.0000000000001959. 10.1097/MD.0000000000001959 PMC5058882 26683910

[24] McKeown RE (2009). The epidemiologic transition: changing patterns of mortality and population dynamics. Am J Lifestyle Med.

[25] Conceição P, Odusola A, Bhorat H, Cornia G. Income inequality trends in sub-Saharan Africa: divergence, determinants, and consequences. 2017.

[26] Anyanwu JC, Erhijakpor AEO, Obi E (2016). Empirical analysis of the key drivers of income inequality in West Africa. Afr Dev Rev.

[27] Bovet P, Ross AG, Gervasoni J-P, Mkamba M, Mtasiwa DM, Lengeler C (2002). Distribution of blood pressure, body mass index and smoking habits in the urban population of Dar Es Salaam, Tanzania, and associations with socioeconomic status. Int J Epidemiol.

[28] Wu F, Guo Y, Chatterji S, Zheng Y, Naidoo N, Jiang Y (2015). Common risk factors for chronic non-communicable diseases among older adults in China, Ghana, Mexico, India, Russia and South Africa: the study on global AGEing and adult health (SAGE) wave 1. BMC Public Health.

[29] Okoduwa SIR, Umar IA, Ibrahim S, Bello F, Ndidi US (2015). Socio-economic status of patients with type 2 diabetes and hypertension attending the Ahmadu Bello University teaching hospital, Zaria, North-West Nigeria. Glob J Health Sci.

[30] Juliet A, Liam S, Leon David A (2007). Hypertension In Sub-Saharan Africa. Hypertension.

[31] Swai ABM, Mclarty DG, Kitange HM, Kilima PM, Tatalla S, Keen N (1993). Low prevalence of risk factors for coronary heart disease in rural Tanzania. Int J Epidemiol.

[32] Olack B, Wabwire-Mangen F, Smeeth L, Montgomery JM, Kiwanuka N, Breiman RF (2015). Risk factors of hypertension among adults aged 35-64 years living in an urban slum Nairobi, Kenya. BMC Public Health.

[33] Arndt C, Hussain MA, Østerdal LP. Effects of food Price shocks on child malnutrition: the Mozambican experience 2008/09. 2012. https://www.wider.unu.edu/sites/default/files/wp2012-089.pdf. 10.1016/j.ehb.2016.03.003 26991234

[34] Van Abeelen AFM, Veenendaal MVE, Painter RC, De Rooij SR, Thangaratinam S, Van Der Post JAM (2012). The fetal origins of hypertension: a systematic review and meta-analysis of the evidence from animal experiments of maternal undernutrition. J Hypertens.

