# Correction to: The prevalence of hypertension and its distribution by sociodemographic factors in Central Mozambique: a cross sectional study

**DOI:** 10.1186/s12889-020-10059-y

**Published:** 2020-12-28

**Authors:** Mika Matsuzaki, Kenneth Sherr, Orvalho Augusto, Yoshito Kawakatsu, Kristjana Ásbjörnsdóttir, Falume Chale, Alfredo Covele, Nelia Manaca, Alberto Muanido, Bradley H. Wagenaar, Ana O. Mocumbi, Sarah Gimbel, the InCoMaS Study Team

**Affiliations:** 1 Department of International Health, Johns Hopkins Bloomberg School of Public Health, Baltimore, MD USA; 2 Department of Global Health, University of Washington, Seattle, WA USA; 3 Health Alliance International, Seattle, WA USA; 4 Department of Community Medicine, Eduardo Mondlane University, Maputo, Mozambique; 5 Department of Epidemiology, University of Washington, Seattle, WA USA; 6 CIOB, University of Washington, Seattle, WA USA; 7 INS, Maputo, Mozambique; 8 Department of Family and Child Nursing, University of Washington, Seattle, WA USA

**Correction to: BMC Public Health 20, 1843 (2020)**

**https://doi.org/10.1186/s12889-020-09947-0**

It was highlighted that in the original article [1] some of the authors Given names and Family names were erroneously interchanged. This Correction article shows the correct author names. The original article has been updated.
