# Supplementary material for: The prevalence of hypertension and its distribution by sociodemographic factors in Central Mozambique: a cross sectional study
Source: BMC Public Health. 2020 Dec 1;20:1843. doi: 10.1186/s12889-020-09947-0 (PMC7709228; doi:10.1186/s12889-020-09947-0)
Supplement: Supplementary file 1 — Additional file 1: Supplemental Material S3. Relevant sections from the InCoMaS 2016 Community Survey Manica and Sofala: (English version). [file 12889_2020_9947_MOESM1_ESM.docx]

**Supplemental Material S3: Relevant sections from the InCoMaS 2016 Community Survey Manica and Sofala: (English version)**

This supplemental material includes questions within the full survey that are related to hypertension and variables included in this paper. The questionnaire was developed in Portugues originally and translated to English for this manuscript.

*The measurements of the blood pressure was measured at three points throughout the interview to have at least two valid measurements that are at least 5 minutes apart.

**IDENTIFICATION**

| **QUESTIONS** | **CODING CATEGORIES** |
| --- | --- |
| GPS COORDINATES | LATITUDE…………………………..............................  LONGITUDE……………………………………………. |
| Province | SOFALA…………………………..............................  MANICA……………………………………………. |
| HOUSEHOLD STUDY CODE | URBAN…………………………………………………...  RURAL…………………………………………………... |
| HOUSEHOLD SELECTED FOR THE FOLLOWING MODULES | Maternal and child health  Children aged 0-4……………………………………..  Children aged 5-14……………………………………  Mother of children under 5………………………......  Other women of childbearing age, 15-49………….. |
|  |  |
|  | Men aged 15 years and older………………………..  Women aged 50 years and older………………....... |

**CHARACTERISTICS OF THE RESPONDENT**

| **QUESTIONS** | **CODING CATEGORIES** |
| --- | --- |
| Note the time | HOURS……………………………...  MINUTES……………………………. |
| In which month and year were you born? | MONTH……………………………...  MONTH NOT KNOWN…………….  YEAR………………………………..  YEAR NOT KNOWN………………. |
| What is your age in years?  COMPARE 201 AND 202 AND CORRECT IF INCONSISTENCIES ARE FOUND | AGE IN YEARS……………………. |
| (NAME) is it male or female? | Man  Women |
| Did you ever attend school? | YES………………………................  NO…………………………………… |
| What was the highest level of schooling you attended? | ILLITERATE………………  LOWER PRIMARY………………  UPPER PRIMARY…………….  LOWER SECONDARY……………  UPPER SECONDARY……………  ELEMENTARY TECHNICIAN…  BASIC TECHNICIAN………………  INTERMEDIATE TECHNICIAN.….  HIGHER………………………….. |
| What was the highest grade / year you completed at this level?  IF SUBJECT DID NOT COMPLETE A FULL GRADE OR YEAR, WRITE '00'. | CLASS / YEAR…………………….. |
| WEIGHT IN KILOGRAMS | KG……………………………………  ABSENT…………………………….  REFUSED…………………………..  OTHER……………………………… |
| HEIGHT IN CENTIMETERS | CM…………………………………...  ABSENT…………………………….  REFUSED…………………………..  OTHER……………………………… |
| BLOOD PRESSURE* | BLOOD PRESSURE (mmHg) 1st measurement: systolic tension  BLOOD PRESSURE (mmHg) 1st measurement: diastolic tension  BLOOD PRESSURE (mmHg) 2nd measurement: systolic tension  BLOOD PRESSURE (mmHg) 2nd measurement: diastolic tension  BLOOD PRESSURE (mmHg) 3rd measurement: systolic tension  BLOOD PRESSURE (mmHg) 3rd measurement: diastolic tension |
| INSTRUCTION ON BLOOD PRESSURE AND BMI OUTCOMES | Mean systolic blood pressure:  If the average systolic TA is above 139 mmHg or if the average diastolic TA is above 89 mmHg, we consider high TA. Please refer the participants to the health unit for evaluation and follow-up.  Mean diastolic pressure:  If the average systolic TA is above 139 mmHg or if the average diastolic TA is above 89 mmHg, we consider high TA. Please refer the participants to the health unit for evaluation and follow-up.  BMI:  If the respondent has a BMI <18.5 - Has low weight for height.  If the respondent has a BMI> = 18.5 and <25 - Has normal weight for height.  If the respondent has a BMI> = 25 and <30 - Is overweight  If the respondent has a BMI> = 30 - Is overweight for height |

**NON-TRANSMISSIBLE DISEASES - ADULTS**

| **QUESTIONS** | **CODING CATEGORIES** |
| --- | --- |
| How often do you have a drink containing alcohol? | NEVER...........................................  ONCE A MONTH OR LESS………  2 TO 4 TIMES A MONTH...............  2 TO 4 TIMES A WEEK…………...  4 OR MORE TIMES A WEEK……. |
| Do you currently smoke cigarettes? | YES………………………................  NO…………………………………… |
| How often do you smoke cigarettes? | DAILY.............................................  WEEKLY........................................  MONTHLY......................................  LESS THAN ONCE A MONTH......  NEVER........................................... |
| Have you ever had your blood pressure measured? | YES………………………................  NO……………………………………  NOT SURE…………………………. |
| Has a health care worker ever told you you have high blood pressure or hypertension? | YES………………………................  NO……………………………………  NOT SURE…………………………. |
| Did you ever have a heart attack, chest pain due to heart disease or a stroke? | YES………………………................  NO……………………………………  NOT SURE…………………………. |

**HOUSEHOLD CHARACTERISTICS**

| **QUESTIONS** | **CODING CATEGORIES** |
| --- | --- |
| What is the main source of water supply used by members of this household for drinking? | Piped water: indoors  Piped water: outside the house but inside the yard  Piped water: at the neighbor's house  Piped water: from a fountain  Well water: protected well  Well water: well NOT protected  Well water: borehole with hand pump  Well water: rainwater  Well water: tank truck  Well water: river / stream / lake / pond water  Well water: bottled / mineral water  Other |
| What kind of bathroom do household members usually use here at home? | Improved traditional latrine  NOT improved latrine  No latrine / Countryside / Defecate in the open  Other |
| Durable goods:  The household has: | Electricity  Radio  Television  Cell phone  Landline  Refrigerator / Freezer |
| What is the main source of energy or fuel that the household uses for cooking? | Electricity  Natural gas  Oil / Paraffin / Kerosene  Mineral coal  Charcoal  Firewood  Animal feces  Food is not cooked in this house  Another |
| MAIN MATERIAL FOR FLOOR CONSTRUCTION  If you have 2 or more materials and it is not obvious which one is the main one, choose all that apply. | Clay  Unpaved earth  Rudimentary wood  Adobe  Parquet or wood flooring  Terrazzo / tiles  Cement  Other TO SPECIFY |
| MAIN ROOF MATERIAL | Without roof  Grass / Thatch / Palm  Zinc Plates  Lusalite plates  Roof tile  Concrete slab  Other |
| Does any member of the household have: | Clock  Bicycle  Motorcycle  Animal-drawn wagon  Car / Truck  Motorboat  none |
