# Supplementary material for: The prevalence of hypertension and its distribution by sociodemographic factors in Central Mozambique: a cross sectional study
Source: BMC Public Health. 2020 Dec 1;20:1843. doi: 10.1186/s12889-020-09947-0 (PMC7709228; doi:10.1186/s12889-020-09947-0)
Supplement: Supplementary file 2 — Additional file 2: Table S1. Cross-sectional associations of elevated blood pressure (systolic blood pressure ≥ 140 mmHg or diastolic blood pressure ≥ 90 mmHg) with risk factors. Table S2. Cross-sectional association of hypertension with risk factors using American Heart Association’s definition (SBP ≥ 130 mmHg or DBP ≥ 80 mmHg). [file 12889_2020_9947_MOESM2_ESM.docx]

**Table S1: Cross-sectional associations of elevated blood pressure (systolic blood pressure≥140mmHg or diastolic blood pressure≥90mmHg) with risk factors.**

|  | Model 1 |  | Model 2 |  |
| --- | --- | --- | --- | --- |
|  | n=2190 |  | n=2985 |  |
|  | OR (95%CI) | p | OR (95%CI) | p |
| Residence |  |  |  |  |
| urban | 1 | -- | 1 | -- |
| rural | 0.65 ( 0.48 to 0.88 ) | 0.006 | 0.84 ( 0.6 to 1.18 ) | 0.317 |
| Age groups |  |  |  |  |
| 20-30 | 1 | -- | 1 | -- |
| 30-40 | 1.4 ( 1.03 to 1.91 ) | 0.034 | 1.38 ( 1.01 to 1.88 ) | 0.043 |
| 40-50 | 2.06 ( 1.42 to 2.99 ) | <0.001 | 1.96 ( 1.35 to 2.86 ) | <0.001 |
| 50-60 | 2.94 ( 1.92 to 4.49 ) | <0.001 | 2.89 ( 1.89 to 4.42 ) | <0.001 |
| 60 and above | 5.69 ( 3.56 to 9.11 ) | <0.001 | 5.65 ( 3.53 to 9.05 ) | <0.001 |
| Gender |  |  |  |  |
| men | 1 | -- | 1 | -- |
| women | 1.23 ( 0.95 to 1.6 ) | 0.12 | 1.15 ( 0.88 to 1.5 ) | 0.297 |
| BMI (kg/m^2^) | 1.06 ( 1.03 to 1.09 ) | <0.001 | 1.05 ( 1.02 to 1.08 ) | 0.001 |
| Education |  |  |  |  |
| primary or less | 1 | -- | 1 | -- |
| secondary | 1.14 ( 0.85 to 1.52 ) | 0.39 | 1.01 ( 0.75 to 1.36 ) | 0.962 |
| above secondary | 1.46 ( 0.84 to 2.53 ) | 0.181 | 1.29 ( 0.74 to 2.24 ) | 0.368 |
| Relative wealth |  |  |  |  |
| above average |  |  | 1.47 ( 1 to 2.15 ) | 0.051 |
| average |  |  | 1 | -- |
| below average |  |  | 0.81 ( 0.55 to 1.17 ) | 0.256 |

Multilevel model was used to account for clustering at household and PSU levels. The confidence intervals were estimated with Wald tests.

All models were adjusted for province.

Reference groups are: rural for residence; male for gender; no for smoking; never for alcohol, above secondary for education.

* no evidence of interaction between residence and education.

Table S2: Cross-sectional association of hypertension with risk factors using American Heart Association’s definition (SBP≥130mmHg or DBP≥80mmHg).

|  | Model 1 |  | Model 2 |  |
| --- | --- | --- | --- | --- |
|  | n=2190 |  | n=2985 |  |
|  | OR (95%CI) | p | OR (95%CI) | p |
| Residence |  |  |  |  |
| urban | 1 | -- | 1 | -- |
| rural | 0.64 ( 0.5 to 0.83 ) | 0.001 | 0.81 ( 0.62 to 1.07 ) | 0.14 |
| Age groups |  |  |  |  |
| 20-30 | 1 | -- | 1 | -- |
| 30-40 | 1.35 ( 1.07 to 1.7 ) | 0.01 | 1.32 ( 1.05 to 1.66 ) | 0.017 |
| 40-50 | 1.74 ( 1.31 to 2.32 ) | <0.001 | 1.69 ( 1.27 to 2.25 ) | <0.001 |
| 50-60 | 2.43 ( 1.7 to 3.48 ) | <0.001 | 2.38 ( 1.66 to 3.41 ) | <0.001 |
| 60 and above | 3.85 ( 2.5 to 5.93 ) | <0.001 | 3.8 ( 2.46 to 5.85 ) | <0.001 |
| Gender |  |  |  |  |
| men | 1 | -- | 1 | -- |
| women | 0.9 ( 0.73 to 1.1 ) | 0.284 | 0.84 ( 0.68 to 1.03 ) | 0.095 |
| BMI (kg/m^2^) | 1.07 ( 1.04 to 1.1 ) | <0.001 | 1.06 ( 1.04 to 1.09 ) | <0.001 |
| Education |  |  |  |  |
| primary or less | 1 | -- | 1 | -- |
| secondary | 1 ( 0.8 to 1.26 ) | 0.971 | 0.89 ( 0.7 to 1.12 ) | 0.306 |
| above secondary | 1.28 ( 0.79 to 2.07 ) | 0.317 | 1.06 ( 0.65 to 1.72 ) | 0.816 |
| Relative wealth |  |  |  |  |
| above average |  |  | 1.33 ( 0.99 to 1.78 ) | 0.062 |
| average |  |  | 1 | -- |
| below average |  |  | 0.71 ( 0.54 to 0.93 ) | 0.014 |

In this analysis, 1538 (37.5%) of the adult participants (35.0% among women; 41.3% among men) met the American Heart Association’s definition of hypertension.
